# Differential Neurodevelopmental Toxicity of Propofol and Fospropofol: Mechanistic Insights and Potential Clinical Implications—A Zebrafish Model Study

**DOI:** 10.3390/ijms27198494

**Published:** 2026-09-23

**Authors:** Zhe Liu, Yingying Deng, Yuanye Gao, Chunyu Jia, Xueqi Zhao, Leyan Tao, Baochang Lai, Xinyan Chen, Yansong Li, Yue Han, Yaomin Zhu

**Affiliations:** 1 Department of Anesthesiology, The First Affiliated Hospital of Xi’an Jiaotong University, Xi’an 710061, China; 2 Department of Physiology and Pathophysiology, School of Basic Medical Sciences, Health Science Center, Xi’an Jiaotong University, Xi’an 710049, China; 3 Cardiovascular Research Center, School of Basic Medical Sciences, Health Science Center, Xi’an Jiaotong University, Xi’an 710061, China

**Keywords:** propofol, fospropofol, zebrafish embryos, neurodevelopmental toxicity

## Abstract

Propofol and fospropofol are used in pediatric surgical anesthesia, but their comparative developmental safety remains uncertain. In this study, zebrafish embryos were exposed to equianesthetic concentrations of propofol or fospropofol. Embryonic survival, hatching, malformation, and post-anesthesia motor behavior were assessed. RNA sequencing and molecular docking were performed to explore potential molecular mechanisms. Fospropofol exhibited lower developmental toxicity than propofol, as shown by the higher survival and hatching rates and a lower malformation rate. Comparative toxicogenomic analysis revealed distinct molecular pathways and candidate targets that may underlie these differential effects. Collectively, these zebrafish data suggest a favorable developmental safety profile for fospropofol and underscore the need for dose optimization and monitoring in pediatric anesthetic management; validation in mammalian models and controlled human studies is further warranted.

## 1. Introduction

Propofol is a widely used intravenous sedative and anesthetic for surgical procedures and intensive care because of its rapid onset and recovery [1,2]. However, accumulating preclinical and clinical evidence suggests potential developmental neurotoxicity [3,4]. Studies indicate that propofol exposure may induce neurodevelopmental and cognitive deficits in infants [5,6], potentially mediated through mechanisms such as dysregulated autophagy, apoptosis, neuroinflammation, mitochondrial dysfunction, calcium overload, and impaired neurotrophic signaling [7]. These findings highlight the need for anesthetic strategies with improved developmental safety.

Fospropofol, a water-soluble propofol prodrug, is converted to active propofol by endothelial alkaline phosphatases [8]. Compared to propofol, it offers advantages including improved solubility, prolonged duration, reduced injection pain, and fewer cardiorespiratory adverse effects [9,10]. Despite these benefits, the impact of fospropofol on children’s neurodevelopment remains unexplored, raising concerns among anesthesiologists regarding its perioperative safety in pediatric populations.

Zebrafish (*Danio rerio*) is a well-established model for developmental neurotoxicity studies [11,12]. Their high fecundity, rapid organogenesis within 5 days post-fertilization, and optical transparency allow for a direct observation of embryogenesis [13]. Zebrafish share more than 80% genetic homology with humans and exhibit conserved neurodevelopmental processes and brain functions [14,15,16]. This model has been used to investigate the neurotoxicity of anesthetics such as ketamine [17], midazolam [18], and propofol [19], and offers advantages for high-throughput mechanistic screening and drug discovery [20,21].

The comparative developmental and neurodevelopmental effects of propofol and fospropofol at concentrations selected to produce comparable anesthetic effects remain incompletely defined. In this study, we used zebrafish embryos to compare the developmental and neurodevelopmental toxicity of these two agents and to assess their differential effects on embryonic survival, morphology, and neural development. We further explored potential molecular mechanisms using transcriptomics and molecular docking. This work was designed as a preclinical, hypothesis-generating investigation; the findings require validation in mammalian models and controlled human studies before clinical inference can be made.

## 2. Results

### 2.1. Fospropofol Exposure Is Associated with Higher Embryonic Survival than Propofol at Equianesthetic Administered Concentrations in Zebrafish

To evaluate developmental toxicity, zebrafish embryos at 8 h post-fertilization (hpf) were exposed to 5–160 µg/mL propofol or fospropofol, and survival was monitored until 6 days post-fertilization (dpf). Both agents caused concentration-dependent mortality, but their potency profiles differed (Figure 1). At 2 dpf, the lowest concentration associated with mortality was 10 µg/mL for propofol and 40 µg/mL for fospropofol (Figure 1A). At 3 dpf, 160 µg/mL fospropofol resulted in approximately 60% survival, whereas propofol at the same concentration caused complete mortality (Figure 1B). At 4 dpf, both 80 and 160 µg/mL fospropofol were associated with reduced survival, whereas the same concentrations of propofol caused complete mortality (Figure 1C). At 5 dpf, 40 µg/mL fospropofol resulted in approximately 35% survival, whereas the same concentration of propofol caused complete mortality (Figure 1D). Under continuous exposure until 6 dpf, 40 µg/mL fospropofol still permitted survival, whereas 20 and 40 µg/mL propofol caused 100% mortality (Figure 1E,F). These observations are consistent with delayed and attenuated toxicity following fospropofol exposure. Together, these data indicate that fospropofol exposure was associated with lower developmental toxicity than propofol in zebrafish embryos. However, because fospropofol is a prodrug, this difference may partly reflect slower or incomplete conversion to active propofol. Concentrations of 20 µg/mL fospropofol and 8 µg/mL propofol, which produced comparable anesthetic effects in the behavioral assay, were therefore selected for subsequent experiments.

### 2.2. Anesthetic Effects of Propofol and Fospropofol in Zebrafish Embryos

To assess anesthetic potency, zebrafish embryos at 24 and 48 h post-fertilization (hpf) were exposed to sublethal concentrations of propofol or fospropofol.

At 24 hpf, anesthesia was defined as the cessation of spontaneous tail coiling. Control embryos exhibited an average of 3.7 coils per minute. Propofol at 2 µg/mL reduced coiling frequency to 0.2 coils/min, and complete anesthesia (0 coils/min) was achieved with 4–8 µg/mL. Fospropofol required higher administered concentrations to achieve comparable effects: 5 and 10 µg/mL reduced coiling to 1.6 and 0.3 coils/min, respectively, and complete anesthesia was observed only at 15–20 µg/mL (Figure 2A,B).

At 48 hpf, anesthesia was evaluated as loss of response to light tactile stimulation. Embryos treated with 8 µg/mL propofol showed no movement in response to touch, whereas responses were observed in the 5 µg/mL propofol group and in the controls (Figure 2B). Complete anesthesia was induced by 20 µg/mL fospropofol (Figure 2C). These results indicate that on an administered-concentration basis, fospropofol required approximately 2.5-fold concentrations than propofol to induce comparable anesthesia in zebrafish embryos.

Recovery time was assessed after the discontinuation of anesthesia at 48 hpf. Embryos anesthetized with 8 µg/mL propofol required approximately 3.5 h to regain spontaneous movement, whereas those exposed to 20 µg/mL fospropofol recovered within 20–25 min (Figure 2D), indicating faster recovery from fospropofol.

In summary, although fospropofol had lower anesthetic potency than propofol, fospropofol exposure was associated with faster recovery. These differences may partly reflect the need for enzymatic conversion of fospropofol to active propofol.

### 2.3. Fospropofol Exposure Is Associated with Less Severe Developmental Abnormalities than Propofol in Zebrafish

Following the assessment of anesthetic potency, sublethal concentrations of 8 µg/mL propofol and 20 µg/mL fospropofol were used to evaluate the effects on embryonic development including malformation, hatching, and heart rate (Figure 3A).

Compared with normal embryos (C1), both treatments were associated with phenotypic abnormalities categorized as mild body curvature (C2), curvature with developmental delay (C3), or severe curvature with delay and additional malformations (C4) (Figure 3B). Approximately 90% of embryos in both treatment groups exhibited the C2 phenotype (Figure 3C). Fospropofol was associated with a lower incidence of severe phenotypes than propofol (fospropofol vs. propofol, C3: 2% vs. 4%; C4: 1% vs. 4%) (Figure 3D), suggesting that fospropofol exposure was associated with milder morphological defects.

Hatching outcomes were then assessed. Hatching was completely inhibited at 2 dpf in both groups (Figure 3E). At 3 dpf, hatching rates were similar between the fospropofol and propofol groups (*p* > 0.05) (Figure 3F). Both treatments transiently reduced embryonic heart rate by approximately 50% at 1 and 2 dpf, with recovery to normal levels by 3 dpf (Figure 3G). Collectively, these results suggest that fospropofol exposure is associated with less severe developmental malformations than propofol.

To investigate the cellular basis for this difference, apoptosis was analyzed (Figure 4A). At 2 dpf, fospropofol-treated embryos exhibited significantly fewer apoptotic cells than those exposed to propofol. At 2 dpf, apoptotic cells were predominantly observed in the head region, including the developing brain, and along the axial midline, including the spinal cord region, with relatively sparse signals in the trunk periphery. By 5 dpf, this difference was no longer significant, and the fospropofol groups maintained minimal apoptosis (Figure 4B,C). These findings suggest that the lower early apoptotic response observed with fospropofol may contribute to its milder teratogenic effects.

### 2.4. Fospropofol Exposure Is Associated with Less Severe Neurodevelopmental Alterations than Propofol in Zebrafish

To evaluate potential neurotoxicity, Tg[Hb9:GFP] [22] zebrafish embryos were treated with 8 µg/mL propofol or 20 µg/mL fospropofol. Motor neuron axon morphology was analyzed by live confocal imaging at 1 and 2 dpf.

At 1 dpf, both treatments reduced axon growth, but the impairment was less severe in the fospropofol group. In the fospropofol group, 90% of larvae exhibited shortened axons, with only 2% classified as severely shortened, compared with 85% and 10%, respectively, in the propofol group (Figure 5A–C). Mean axon length was greater in the fospropofol group (83.4 ± 3.1 µm) than in the propofol group (72.7 ± 4.2 µm), although both were reduced relative to the controls (101.3 ± 3.5 µm) (Figure 5D). By 2 dpf, axon length remained more preserved with fospropofol (127.1 ± 4.25 µm) than with propofol (120.8 ± 5.03 µm), relative to the controls (137.3 ± 7.03 µm) (Figure 5D–F). Although abnormal branching increased in both groups, the proportion of larvae with abnormal branching was lower in the fospropofol group (5%) than in the propofol group (15%) (Figure 5E–H). These findings suggest that fospropofol exposure was associated with less severe disruption of motor neuron outgrowth and morphology than propofol.

To further investigate potential effects on neural development, SOX2 expression, a marker of neural stem and progenitor cells, was examined by immunofluorescence. The reduction in SOX2 expression was less pronounced in the fospropofol group than in the propofol group (*p* < 0.05 vs. the control) (Figure 5H–J), suggesting a less pronounced effect on neural progenitor cells.

Together, these results suggest that fospropofol exposure was associated with less severe neurodevelopmental alterations than propofol in this zebrafish model. However, these analyses were limited to Hb9-positive motor neurons and SOX2 expression at 5 dpf; other neuronal subtypes and earlier time points were not evaluated. In addition, because fospropofol is a prodrug, these differences may partly reflect slower or incomplete conversion to active propofol.

### 2.5. Transcriptomic Analysis of the Differential Effects of Propofol and Fospropofol in Zebrafish

To investigate the molecular mechanisms underlying the differential effects of propofol and fospropofol, transcriptome sequencing was performed on 2 dpf zebrafish embryos divided into three groups: the control, propofol-treated (8 µg/mL), and fospropofol-treated (20 µg/mL) groups. Principal component analysis (PCA) and Pearson correlation coefficients confirmed clear separation among the experimental groups (Figure S1A–C). A hierarchical clustering heatmap further illustrated distinct gene expression patterns across groups (Figure S1D).

Gene Ontology (GO) enrichment analysis revealed significant alterations in biological processes, cellular components, molecular functions, and related signaling pathways in both treatment groups relative to the controls. Regarding cellular components, both treatments affected terms related to lysosomes, the extracellular region, and the extracellular space. Notably, propofol exposure was associated with the enrichment of terms related to myeloid cell differentiation and neurodevelopment, whereas these terms were not significantly enriched in the fospropofol group (Figure S2A,B). The Kyoto Encyclopedia of Genes and Genomes (KEGG) pathway analysis indicated that the amino acid metabolism, lysosome, and phagosome pathways were significantly modulated in both treatment groups relative to the controls (Figure 6A,B). Propofol exposure was associated with the enrichment of pathways such as ECM–receptor interaction and cardiac muscle contraction, and with altered expression of genes involved in neurodevelopmental processes including neuronal differentiation (*neurod2*, *pou4f3*) [23,24], axon guidance (*cntn1b*, *cntnap1*) [25,26], and synapse formation (*sv2ba*, *syt5b*) [27,28]. The visual system, specifically photoreceptor development and function (*rpgrip1*) [29], was also a sensitive target of propofol toxicity. Mechanisms underlying propofol’s developmental toxicity may involve induced oxidative stress and disruption of drug-metabolizing enzyme systems (e.g., *gstp2* activation) [30,31,32] (Figure 6C). Fospropofol treatment had several different impacts on pathway enrichment, such as with its effect observed in vascular smooth muscle contraction, and less effects on the phagosome and lysosome (Figure 6B). Altered expression of transcription factors critical for early embryonic patterning (e.g., *tbxta* [33] and *foxa3* [34]) was observed (Figure 6D).

Gene set enrichment analysis (GSEA) was further performed to compare the pathway enrichment patterns between the propofol- and fospropofol-treated groups (Figure 6E,F). The enrichment score (ES) for the phagosome pathway was higher in the propofol group than in the fospropofol group, suggesting a stronger perturbation by propofol. Notably, the upregulation of the apoptosis and autophagy pathways was observed only in the propofol group, consistent with the AO staining results (Figure 4), suggesting that propofol may trigger more pronounced programmed cell death, an effect not prominent with fospropofol. Furthermore, both the propofol and fospropofol groups exhibited the upregulation of genes involved in the negative regulation of axon extension, consistent with the observed axonal changes (Figure 5A,E), suggesting altered regulation of axonal development. These results suggest that the phagosome pathway may contribute to the differential effects of propofol and fospropofol, although its enrichment score was higher in the propofol group. However, because fospropofol is a prodrug, these transcriptomic differences may partly reflect differences in bioconversion.

### 2.6. Comparative Toxicogenomic Analysis Identifies Distinct Molecular Pathways and Candidate Targets for Propofol and Fospropofol

To investigate the molecular basis for their differential effects, we constructed protein–protein interaction (PPI) networks using differentially expressed genes (DEGs) from zebrafish embryos converted to human orthologs. Analysis revealed 444 DEGs common to both treatment groups, with 124 DEGs detected only in the propofol group and 295 only in the fospropofol group relative to the controls (Figure 7A). Because the DEGs were identified relative to the controls, these gene sets indicate distinct response patterns. Network topology analysis of the propofol-associated PPI network highlighted candidate targets including FN1, STAT1, CSF3, and several UGT1A isoforms (Figure 7B; Table S1). The fospropofol-associated network included distinct candidate targets such as PRKACB, PRKACA, and PRKACG, alongside shared targets including FN1 and UGT1A1 (Figure 7C; Table S2). The larger number of DEGs and the more extensive network in the fospropofol group should not be interpreted as greater toxicity; these changes may reflect adaptive or compensatory responses. In addition, different degree thresholds were used for network visualization in Figure 7B,C, and these thresholds do not support direct topological comparisons between the two networks.

Fibronectin 1 (FN1) emerged as a central hub in both networks, suggesting that it may be involved in the responses to both anesthetics; however, PPI hub status does not establish FN1 as a direct drug target. Molecular docking predicted that both anesthetics may bind FN1 in silico, with different predicted binding energies: propofol had a predicted binding energy of −17.41 kJ/mol, whereas fospropofol showed a stronger predicted interaction at −35.80 kJ/mol (Figure 7D,E). These docking results are hypothesis-generating and require experimental validation, such as surface plasmon resonance or cellular thermal shift assays. The predicted differential binding to FN1, a protein involved in cell adhesion and signaling, may contribute to the observed differences in developmental toxicity [35].

The candidate target profiles suggest potentially divergent mechanisms. Propofol-specific candidate targets, such as STAT1 [36] and CSF3 [37], implicate inflammatory and immune pathways, consistent with its broader toxicity profile. In contrast, fospropofol-unique candidate targets such as PRKACB, PRKACA, and PRKACG (protein kinase A subunits) point toward the modulation of intracellular signaling cascades [38,39,40]. Shared candidate targets, including several UGT enzymes such as UGT1A1, suggest common involvement in drug metabolism and detoxification pathways [41]. The different predicted binding affinities for the common hub FN1, together with agent-specific candidate target sets, provide a mechanistic hypothesis for the observed differences in developmental toxicity. Furthermore, these analyses were designed to identify toxicity-associated pathways and candidate targets from transcriptomic data, rather than to identify the primary pharmacological targets of anesthesia, such as GABAA receptors.

## 3. Discussion

In this study employing the zebrafish model, we characterized the differential developmental toxicity profiles of propofol and fospropofol at equianesthetic administered concentrations. Our findings suggest that fospropofol exposure is associated with a more favorable developmental safety profile than propofol in this model. However, because fospropofol is a prodrug requiring enzymatic conversion to active propofol, this difference may partly reflect slower or incomplete bioconversion rather than an intrinsically safer active moiety.

At the phenotypic level, the reduced developmental toxicity of fospropofol was evident in several critical parameters. We observed significantly lower mortality rates, a lower incidence of severe malformations such as spinal curvature and pericardial edema, and a higher hatching rate in embryos exposed to fospropofol. Crucially, the assessment of neurodevelopmental endpoints revealed a markedly milder impact of fospropofol on motor neuron outgrowth and morphology, with less pronounced reductions in axon length and a lower propensity for aberrant branching. Similarly, the expression of SOX2, a critical marker for neural progenitor cells, was better preserved following fospropofol exposure [42]. These observations collectively strengthen the narrative that fospropofol contributes to a less disruptive developmental environment for the nascent nervous system.

Transcriptomic and bioinformatic analyses further elucidated the distinct molecular landscapes activated by each drug. While both compounds affected pathways related to lysosome function and extracellular matrix organization, the anesthetics also perturbed neurodevelopmental processes, including neuronal differentiation [23,24], axon guidance [25,26], and synaptic formation [27,28]. Furthermore, the enhanced activation of the apoptosis and autophagy pathways observed with propofol underscores its capacity to induce more widespread cellular stress and programmed cell death during critical developmental windows, a mechanism largely mitigated by fospropofol.

A central finding of this study is that Fibronectin 1 (FN1) may act as a common target differentially engaged by both anesthetics. However, this study has limitations: molecular docking only generates a testable hypothesis rather than definitive evidence of biological binding, so experimental validation (e.g., surface plasmon resonance or cellular thermal shift assays) is required in the future. Through comparative toxicogenomic analysis and molecular docking, we demonstrated that although both drugs show potential binding affinity in silico to FN1—a key extracellular matrix protein involved in cell adhesion and signaling [35]. Fospropofol exhibited a stronger binding affinity (−35.796 kJ/mol) than propofol (−17.4098 kJ/mol). Interestingly, propofol-induced injection site pain is not observed with fospropofol, suggesting that fospropofol probably does not activate the irritant receptor, transient receptor potential 1 (TRPA1) [43]. The phosphorylated structure of fospropofol abolishes its capacity to activate TRPA1 while concurrently conferring a greater potential to bind FN1. This difference in interaction may lead to distinct downstream signaling perturbations, potentially explaining the milder neurodevelopmental toxicity associated with fospropofol. Furthermore, propofol-specific upregulation of genes such as STAT1 [36] and CSF3 [37] implies involvement in immune and inflammatory responses, whereas fospropofol appears to preferentially affect pathways related to vascular smooth muscle contraction and protein kinase A signaling [38,39,40].

Our PPI and molecular docking analyses were designed to identify toxicity-associated pathways and candidate targets from transcriptomic data, rather than to identify the primary pharmacological targets of anesthesia. Propofol exerts anesthetic effects primarily through positive allosteric modulation of γ-aminobutyric acid type A (GABAA) receptors, and fospropofol, as a prodrug, is expected to act through the same pathway after conversion. FN1 emerged as a shared hub from an unbiased PPI analysis, reflecting its centrality in the differential gene expression networks rather than a role as a direct anesthetic receptor. This does not exclude the involvement of GABAA receptors or other classical targets, such as NMDA or glycine receptors, in the observed neurodevelopmental effects. However, our dataset cannot dissect the relative contributions of direct receptor modulation versus downstream transcriptional responses. Future studies using receptor-specific antagonists or genetic models could clarify this relationship.

From a translational perspective, fospropofol may be associated with a more favorable developmental safety profile in preclinical models. Its lower embryotoxicity and neurotoxicity, together with faster recovery and reduced injection pain, suggest that fospropofol warrants further investigation as a potential alternative to propofol for prolonged sedation or anesthesia in pediatric populations. However, these are preclinical observations and do not constitute clinical evidence. Propofol remains a well-established anesthetic, and its potential for developmental neurotoxicity in immature brains, demonstrated primarily in animal models, underscores the need for vigilance and dose optimization. Our data support a further evaluation of fospropofol in mammalian systems, but clinical recommendations await human data. This study also highlights the utility of zebrafish for preclinical neurotoxicity screening, given its genetic and physiological homology to humans [44,44,45] and its suitability for high-resolution live imaging and large-scale omics analyses [46,47,48,49,50].

### 3.1. Translational Limitations and Clinical Caveats

All findings were obtained solely from zebrafish embryos. Zebrafish and humans differ in drug metabolism, pharmacokinetics, blood–brain barrier characteristics, and the temporal sequence of neurodevelopment [18,21]. Although the administered concentrations were selected to produce comparable anesthetic effects, they may not reflect human clinical exposure. Therefore, these results should be interpreted as hypothesis-generating preclinical evidence and do not support any adjustment to current clinical practice. Validation in mammalian models, such as rodents and non-human primates, and ultimately well-controlled pediatric studies, are essential before any clinical recommendation can be made regarding the preferential use of fospropofol in children.

### 3.2. Limitation

Apoptotic signals were predominantly localized to the head and axial midline regions; however, quantitative regional segmentation of AO staining was not performed. Such analysis would strengthen the assessment of organ-specific toxicity and is planned for future studies.

Motor neuron analysis was restricted to Hb9-positive motor neurons. Other neuronal populations, including interneurons, sensory neurons, and photoreceptor neurons, were not assessed. Although transcriptomic data revealed effects on genes associated with photoreceptor development (rpgrip1), neuronal differentiation (neurod2, pou4f3), and axon guidance (cntn1b, cntnap1), direct histological assessment of these populations is required for a comprehensive neurotoxicity profile.

SOX2 immunofluorescence was performed only at 5 dpf. Although transcriptomic data at 2 dpf suggest the early downregulation of SOX2 mRNA, protein-level analysis at earlier time points is needed to establish the timing of neural progenitor damage versus recovery. This temporal gap is a limitation and a priority for follow-up studies.

Molecular docking predicted potential anesthetic–FN1 interactions, but molecular dynamics simulation would be required to assess binding stability in a dynamic environment. Such simulations were beyond the scope of this study, and the docking results are therefore presented as hypothesis-generating rather than confirmatory. In addition, because fospropofol is a prodrug, the extent of its conversion to active propofol in zebrafish embryos was not measured; future studies should quantify active propofol concentrations in zebrafish tissues.

## 4. Materials and Methods

### 4.1. Zebrafish Husbandry

The AB strain and Tg[Hb9:GFP] [22] transgenic zebrafish were obtained from the China Zebrafish Resource Center (CZRC, located in Wuhan, China). The fish were housed under standard laboratory conditions, with water temperature maintained at 28.5 °C and a 14 h:10 h light–dark cycle. Embryos were collected and cultivated in Petri dishes within a biochemical incubator set at 28.5 °C. No additional control was implemented regarding housing locations or measurement sequences; however, all groups were treated synchronously under identical environmental conditions. All experimental procedures were conducted in accordance with the ethical guidelines provided by the CZRC and approved by the Animal Experiment Ethics Committee of Xi’an Jiaotong University.

### 4.2. Embryo Intervention

All experiments were performed with three independent biological replicates, each derived from a separate spawning event. For each replicate, 50 embryos were placed per 90-mm dish, and three technical replicate dishes were used per treatment group. Each dish contained 50 embryos in 30 mL of medium. Embryos were assigned randomly to either the control or propofol groups. The sample size was determined based on the conventional criteria used in previously published studies of zebrafish neurodevelopmental toxicity in this field.

### 4.3. Drugs

Propofol (AstraZeneca, Wilmington, DE, USA, 10 mg/mL lipid emulsion) and fospropofol disodium (Yichang Humanwell, Yichang, China, ≥99% purity by HPLC, dissolved in sterile saline) were used as commercial clinical-grade formulations, and the vehicle controls were blank lipid emulsion and normal saline, respectively, administered at matching volumes.

### 4.4. Developmental Toxicity Assay

Developmental toxicity in zebrafish embryos and larvae was assessed based on spinal curvature, pericardial edema, mild pigmentation, and yolk sac edema. At each observation time point, survival and hatching rates were calculated as percentages based on counts of live, damaged, and hatched embryos per dish (out of 50 embryos). Heart rate was measured by counting heartbeats per minute via slow-motion video playback. For axon length measurement, larvae were explored in propofol or fospropfol. Manual dechorionation using forceps was performed under a stereomicroscope for all unhatched embryos.

### 4.5. In Situ AO Assay

Zebrafish embyoes were cultured in E3 buffer supplemented with 0.003% PTU (1-phenyl-2-thiourea, Sigma-Aldrich, St. Louis, MO, USA) to inhibit melanin formation. Apoptosis was detected using a commercial Acridine Orange (AO, PS0063, Psaitong, Tianjin, China). Images were obtained with a stereo fluorescence microscope (M165FC, Leica, Germany) and processed using ImageJ software (version 1.54, NIH, Bethesda, MD, USA).

### 4.6. Immunofluorescence on Zebrafish Paraffin Sections

Zebrafish larvae were fixed in 4% paraformaldehyde (PFA) overnight at 4 °C. Samples were embedded in paraffin and sectioned at a thickness of 5 µm using a rotary microtome. Sections were mounted on glass slides, dewaxed in xylene, and rehydrated through a descending ethanol gradient to phosphate-buffered saline (PBS). Antigen retrieval was performed by incubating sections in citrate buffer (pH 6.0) at 85 °C for 15 min. Sections were washed in PBS and incubated with appropriate fluorophore-conjugated antibodies. Rabbit anti-SOX2 primary antibody (GTX124477, dilution 1:100, overnight incubation at 4 °C), blocking with 5% normal goat serum for 1 h. After final washes, slides were coverslipped using anti-fade mounting medium. Images were acquired using a laser scanning confocal microscope (ZEISS, Jena, Germany).

### 4.7. Transcriptomic Sequencing

At 48 h post-fertilization (hpf), zebrafish from the control, propofol, and fospropofol groups were collected for transcriptomic profiling. Each group consisted of 50 zebrafish, with 4 biological replicates per condition. Total RNA was extracted and subjected to high-throughput RNA sequencing (RNA-Seq) using the Illumina NovaSeq™ 6000 platform in PE150 mode, performed by LC Bio Technology Co., Ltd. (Hangzhou, China). Differential gene expression (DEG) analysis between the treatment and control groups was conducted using a threshold of an adjusted *p*-value < 0.05. Significantly altered genes were subsequently analyzed for functional enrichment in Gene Ontology (GO) terms and Kyoto Encyclopedia of Genes and Genomes (KEGG) pathways, with a corrected *p*-value < 0.05 considered significant. Leading-edge analysis further highlighted core genes contributing to enrichment, the expression patterns of which were visualized [46,51,52]. All transcriptomic analyses were based on RNA-Seq data, which were generated with four biological replicates per group and subjected to strict quality control (PCA and Pearson correlation, Figure S1A–C).

### 4.8. Protein–Protein Interaction (PPI) Network

Differentially expressed genes (DEGs) from the RNA-Seq analysis were selected as potential targets related to propofol and fospropofol exposure. Zebrafish DEGs were mapped to human orthologs using the Ensembl database to enable cross-species analysis. These human gene counterparts were submitted to the STRING database to construct a PPI network. Node degree was used as a key metric to evaluate the centrality of target proteins within the network [53].

### 4.9. Molecular Docking

The ligand structures of propofol and fospropofol were sourced from PubChem. The crystal structure of FN1 (PDB ID: 2cg7) was retrieved from the RCSB PDB database. The interactions and binding poses were visualized using PyMOL (version 2.6.0a0) [53,54].

### 4.10. Statistical Analysis

Data are presented as the mean ± SD. One-way ANOVA followed by Dunnett’s post hoc test was applied for comparisons between treatment groups and the control when the assumptions were met. Significance levels were defined as * *p* < 0.05, ** *p* < 0.01, *** *p* < 0.001, and **** *p* < 0.0001. All analyses and graphing were performed using GraphPad Prism version 8.0.

## 5. Conclusions

This study integrated phenotypic evaluation, transcriptomic profiling, and molecular docking to compare the developmental and neurodevelopmental effects of fospropofol and propofol in zebrafish. Fospropofol exposure was associated with a more favorable developmental safety profile than propofol in this preclinical model. FN1 was identified as a common hub with differential predicted binding affinity, providing a mechanistic hypothesis for the observed differences. These findings provide a rationale for future investigations into whether fospropofol may offer a favorable safety profile in pediatric anesthesia. However, given the preclinical nature of the zebrafish model, direct clinical translation is not warranted. Further validation in mammalian systems and controlled human studies is required before any clinical recommendation can be made. The observed differences in this model highlight the importance of continued dose optimization and monitoring in pediatric anesthetic management. Future studies should validate these findings in mammalian systems and explore targeted interventions to optimize anesthetic efficacy while maximizing neurodevelopmental protection.

## Figures and Tables

**Figure 1:**
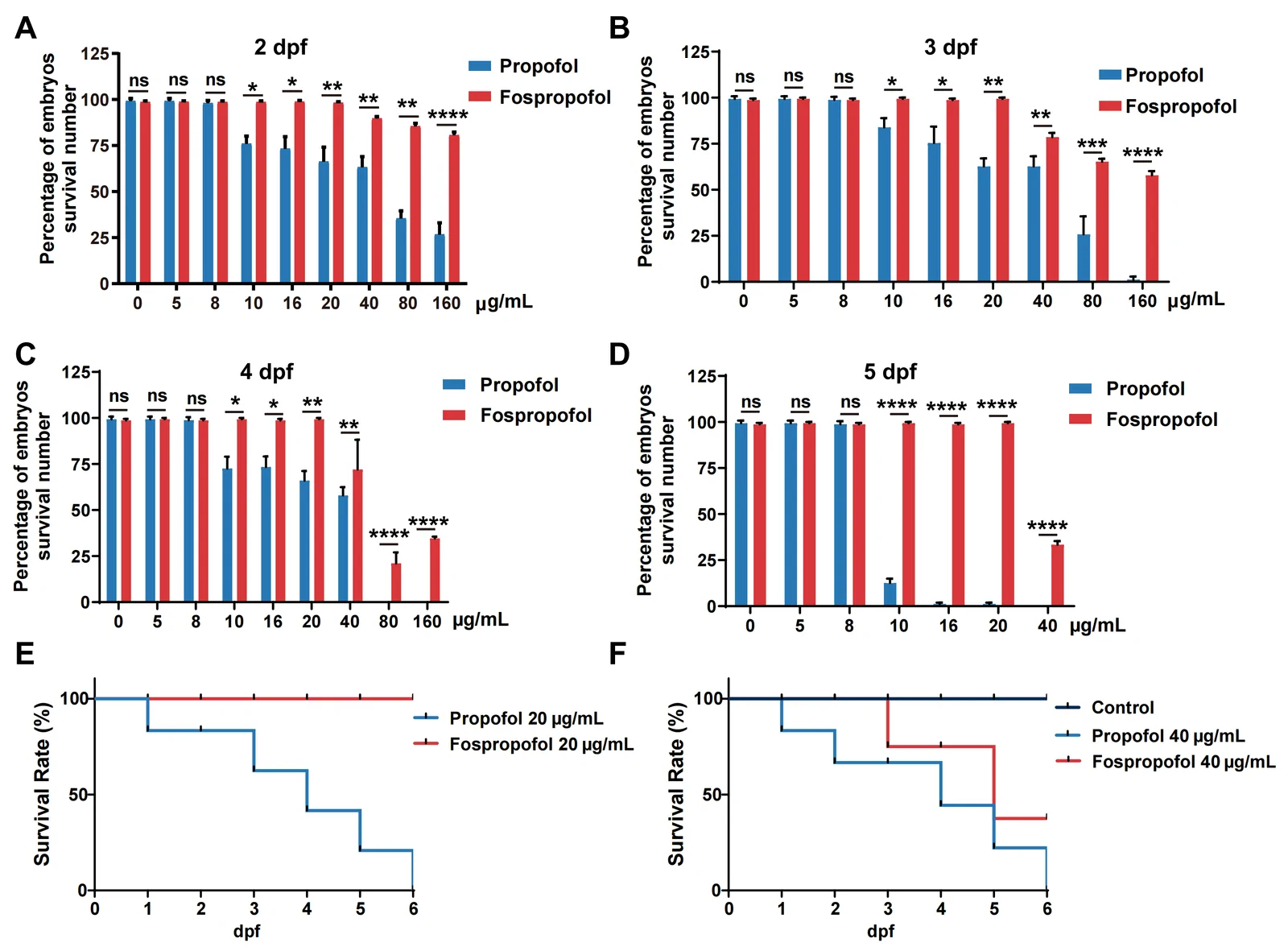
Survival of zebrafish embryos exposed to propofol or fospropofol. (**A**–**D**) Survival rates at 2, 3, 4, and 5 dpf after exposure to different concentrations. (**E**,**F**) Kaplan–Meier survival curves from 2 to 5 dpf for embryos exposed to 20 µg/mL (**E**) and 40 µg/mL (**F**) propofol or fospropofol. Data are the mean ± SD. One-way ANOVA followed by Dunnett’s test: * *p* < 0.05, ** *p* < 0.01, *** *p* < 0.001, **** *p* < 0.0001. ns: not significant.

**Figure 2:**
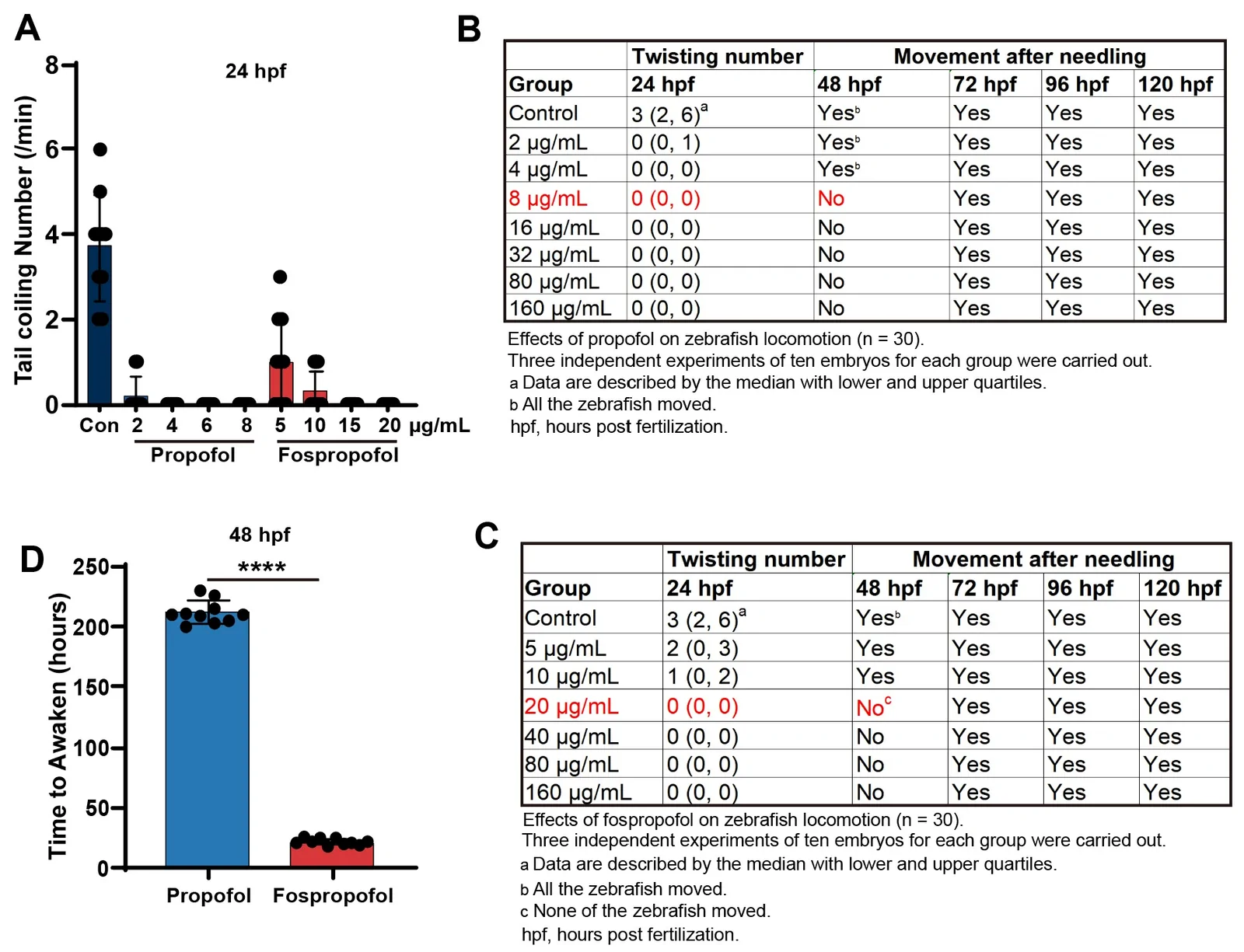
Anesthetic effects of propofol and fospropofol on early zebrafish embryos. (**A**) Anesthesia at 24 hpf, defined as zero body coiling movements per minute. Control embryos averaged 3.7 coils/min. Propofol at 4–8 µg/mL and fospropofol at 15–20 µg/mL induced complete anesthesia. (**B**,**C**) Anesthesia at 48 hpf was defined as absence of response to needle touch. Propofol at 8 µg/mL and fospropofol at 20 µg/mL induced complete anesthesia. (**D**) Recovery time after anesthesia at 48 hpf. Data are the mean ± SD. One-way ANOVA followed by Dunnett’s test: **** *p* < 0.0001 vs. control.

**Figure 3:**
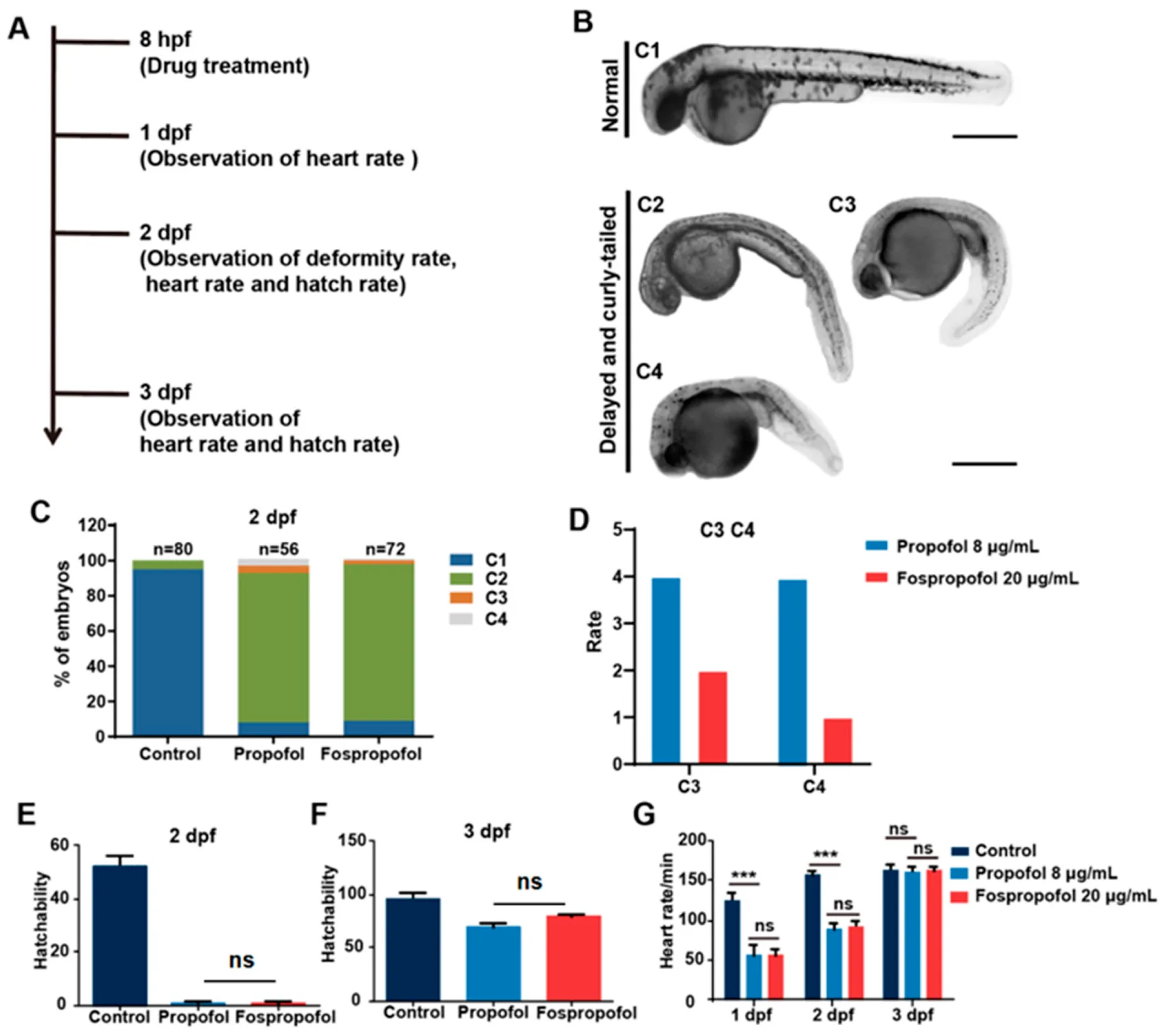
Morphological and physiological assessments of zebrafish embryos after propofol or fospropofol exposure. (**A**) Timeline of treatment and measurement. (**B**) Representative images at 2 dpf. C1, normal; C2, mild body axis curvature; C3, curvature with developmental delay; C4, curvature, delay, and malformation. Scale bar: 0.5 mm. (**C**) Proportion of C1–C4 morphologies at 2 dpf. (**D**) Proportion of C3 and C4 morphologies in propofol and fospropofol groups. (**E**) Hatching rates at 2 dpf. (**F**) Hatching rates at 3 dpf. (**G**) Heart rates at 1, 2, and 3 dpf. Data are the mean ± SD. One-way ANOVA followed by Dunnett’s test: *** *p* < 0.001. ns: not significant.

**Figure 4:**
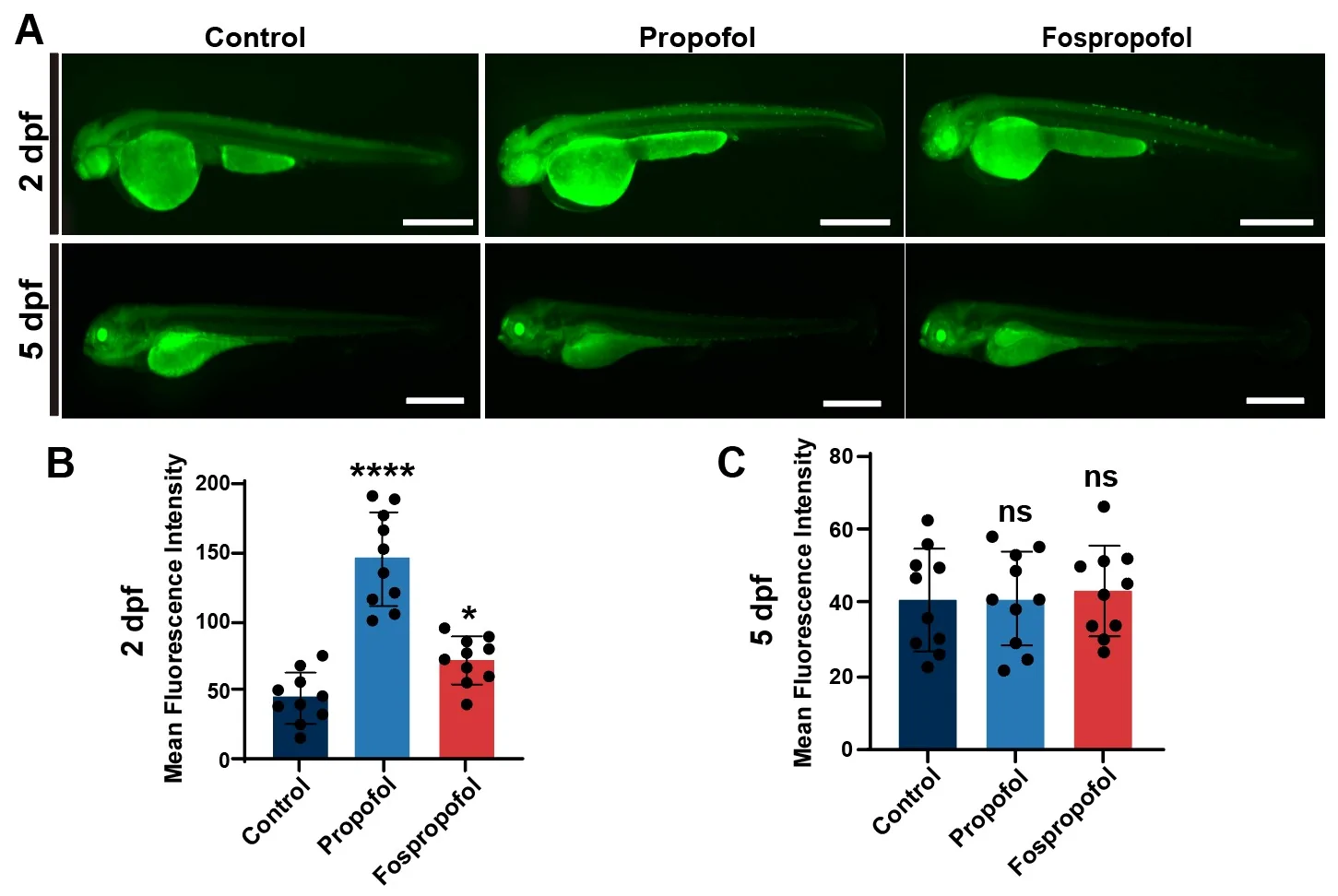
Acridine orange (AO) staining in the control, propofol-, and fospropofol-exposed zebrafish. (**A**) Representative whole-mount images at 2 and 5 dpf. Scale bars: 0.5 mm at 2 dpf; 1 mm at 5 dpf. (**B**,**C**) Quantification of AO fluorescence at 2 dpf (**B**) and 5 dpf (**C**). Data are the mean ± SD. One-way ANOVA followed by Dunnett’s test: * *p* < 0.05, **** *p* < 0.0001 vs. the control. ns: not significant.

**Figure 5:**
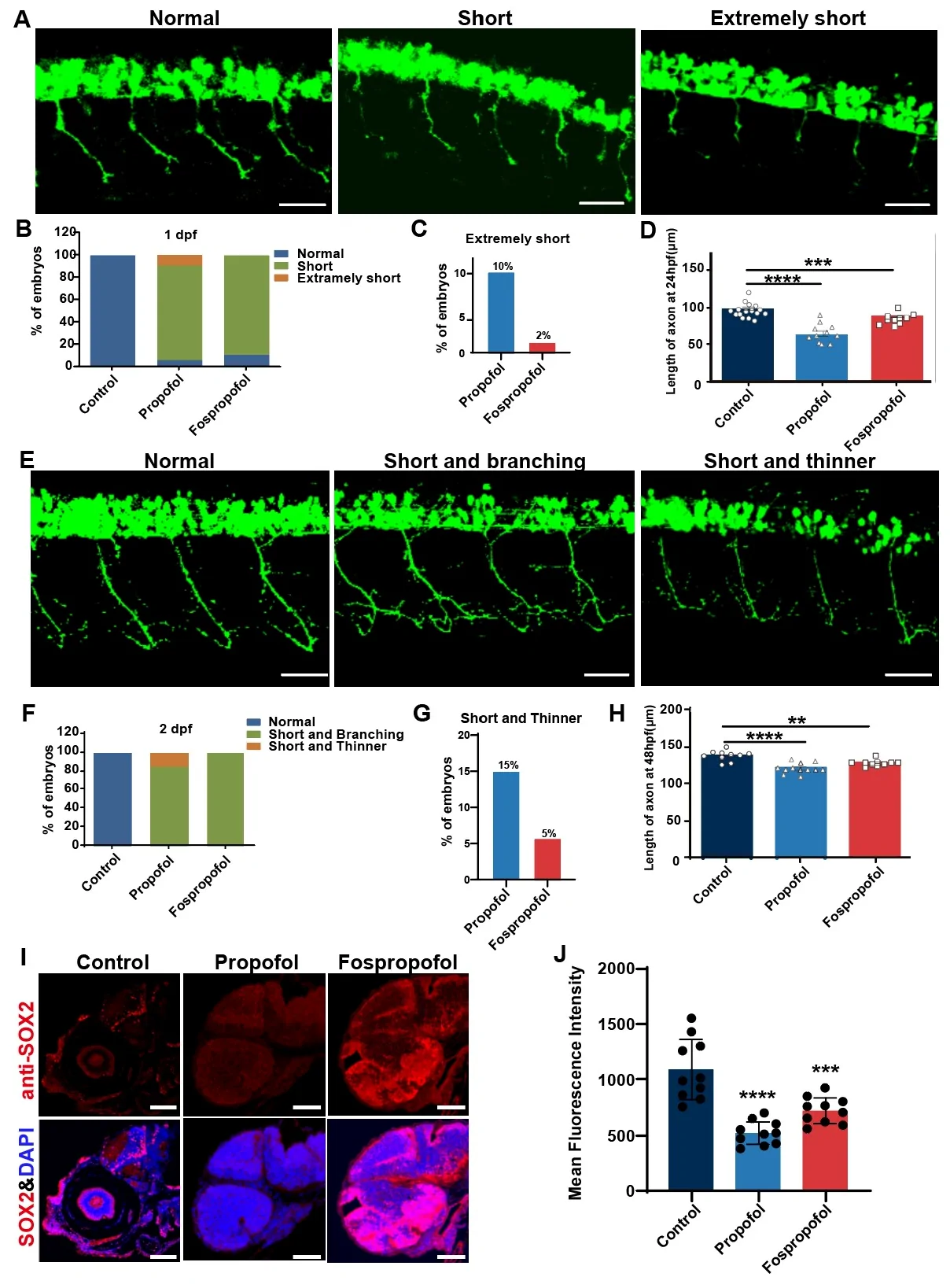
Effects of propofol and fospropofol on motor neuron development and SOX2 expression. (**A**,**B**) Representative images and classification of motor neuron axon morphology in Tg[Hb9:GFP] zebrafish at 1 dpf. Axons were classified as normal, short, or extremely short. Scale bar: 20 µm. (**C**) Proportion of extremely short axons at 1 dpf. (**D**) Axon length at 1 dpf. (**E**,**F**) Representative images and classification at 2 dpf. Axons were classified as normal, branching, or thinner and branching. Scale bar: 30 µm. (**G**) Proportion of thinner and branching axons at 2 dpf. (**H**) Axon length at 2 dpf. (**I**) Representative SOX2 immunostaining of paraffin sections at 5 dpf. Scale bar: 50 µm. (**J**) Quantification of SOX2 expression at 5 dpf. Data are the mean ± SD. One-way ANOVA followed by Dunnett’s test: ** *p* < 0.01, *** *p* < 0.001, **** *p* < 0.0001 vs. the control.

**Figure 6:**
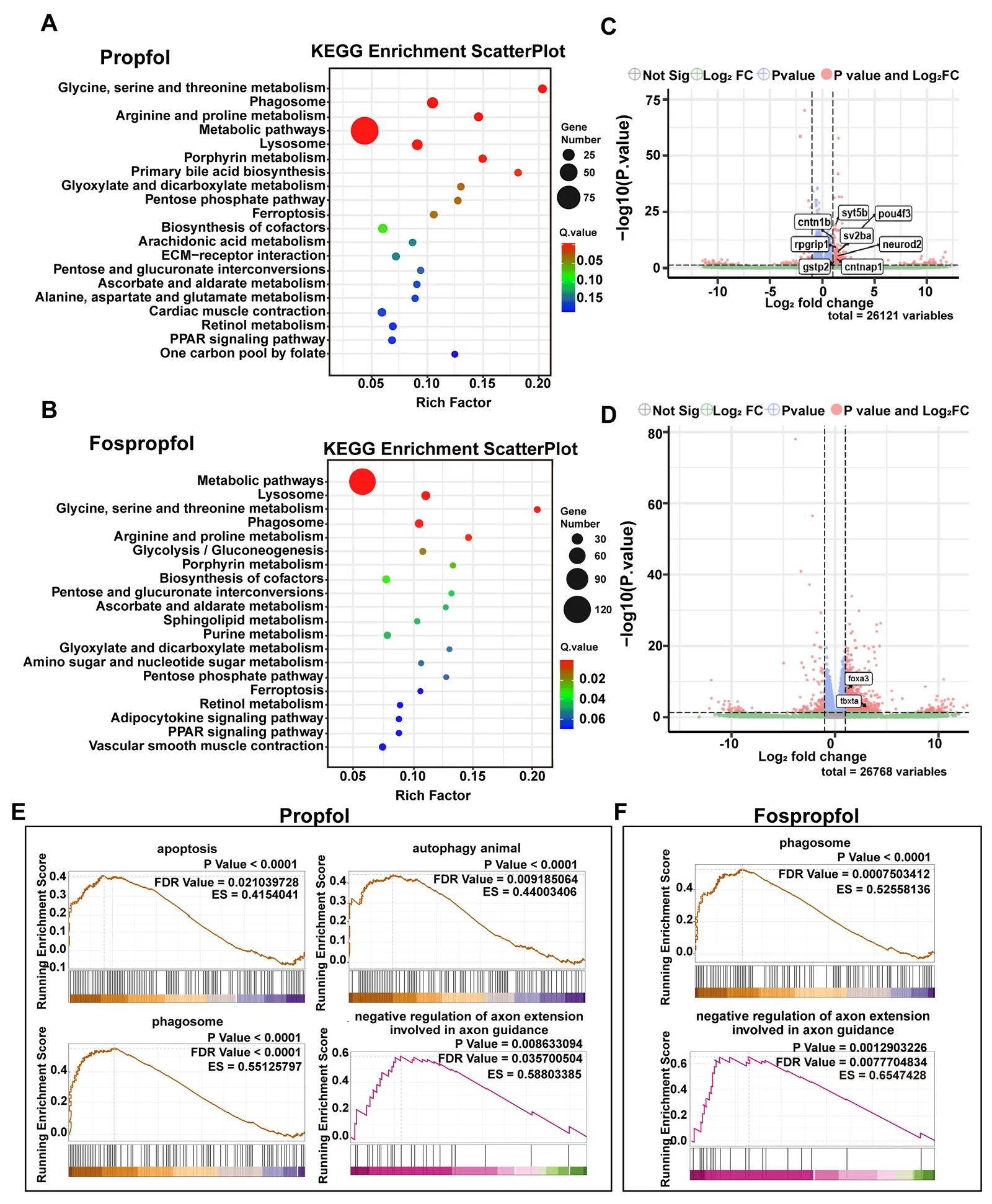
Molecular mechanisms underlying propofol- and fospropofol-induced effects. (**A**,**B**) KEGG enrichment analysis for propofol (**A**) and fospropofol (**B**). (**C**,**D**) Volcano plots of differentially expressed genes (DEGs) associated with morphological changes induced by propofol (**C**) and fospropofol (**D**). (**E**,**F**) Gene set enrichment analysis (GSEA) plots for propofol (**E**) and fospropofol (**F**). Enrichment scores (ES) and false discovery rate (FDR) values are shown.

**Figure 7:**
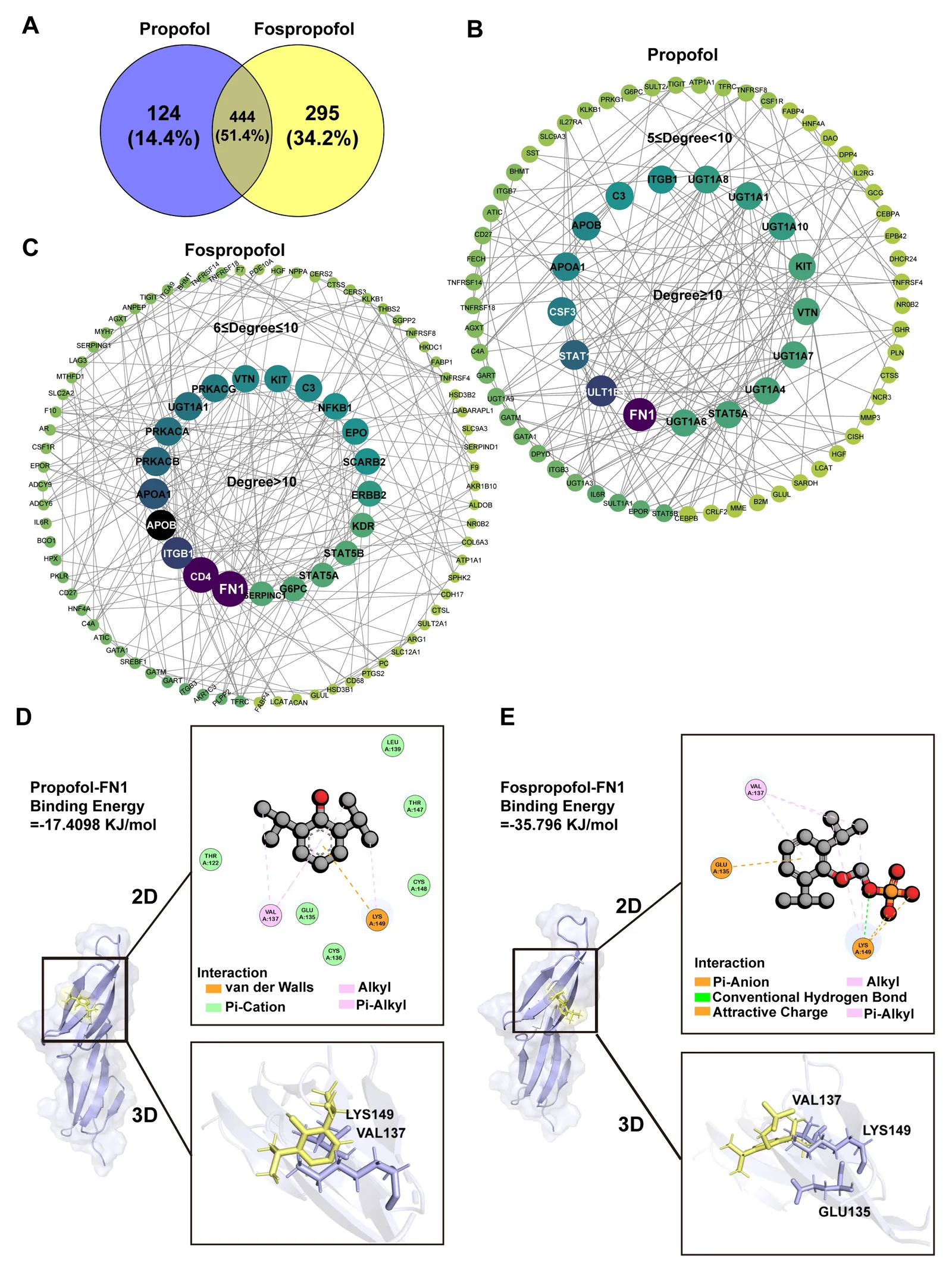
Comparative toxicogenomic analysis of DEGs and PPI networks for propofol and fospropofol, with molecular docking to FN1. (**A**) Venn diagram of DEGs after conversion to human orthologs. (**B**) PPI network from propofol-group DEGs, highlighting genes with degree ≥ 5. (**C**) PPI network from the fospropofol-group DEGs, highlighting genes with degree ≥ 6. Different degree thresholds were used only for visualization and do not support direct topological comparisons. (**D**,**E**) Molecular docking of propofol (**D**) and fospropofol (**E**) with FN1. Predicted binding energies are shown. Docking results are in silico predictions and require experimental validation.

## Data Availability

The original contributions presented in this study are included in the article/Supplementary Materials. Further inquiries can be directed to the corresponding authors.

## Supplementary Materials

The following supporting information can be downloaded at: https://www.mdpi.com/article/10.3390/ijms27198494/s1.
