# Supplementary material for: Differential Neurodevelopmental Toxicity of Propofol and Fospropofol: Mechanistic Insights and Potential Clinical Implications—A Zebrafish Model Study
Source: Int J Mol Sci. 2026 Sep 23;27(19):8494. doi: 10.3390/ijms27198494 (PMC13665160; doi:10.3390/ijms27198494)
Supplement: Supplementary file 1 [file ijms-27-08494-s001.zip › ijms-4505691-supplementary.pdf]

Supplementary Materials

Table S1. Core targets screened from the propofol PPI network.

Gene Name	Degree
FN1	24
SULT1E1	19
STAT1	16
CSF3	14
APOA1	13
APOB	13
C3	12
ITGB1	12
UGT1A10	11
UGT1A1	11
UGT1A8	11
VTN	10
STAT5A	10
UGT1A6	10
UGT1A4	10
UGT1A7	10
KIT	10
IL6R	9
EPOR	9
STAT5B	9
SULT1A1	9
GART	8
GATM	8
GATA1	8
DPYD	8
ITGB3	8
UGT1A3	8
UGT1A9	8
AGXT	7
BHMT	7
ATIC	7
C4A	7
FECH	7
CD27	7
TNFRSF14	7
TNFRSF18	7
ITGB7	7
G6PC	6
TFRC	6
KLKB1	6
ATP1A1	6
PRKG1	6
IL27RA	6
TNFRSF8	6
TIGIT	6
SST	6
CSF1R	6

SULT2A1	6
SLC9A3	6
DAO	5
SARDH	5
HNF4A	5
B2M	5
LCAT	5
NR0B2	5
GLUL	5
PLN	5
NCR3	5
TNFRSF4	5
CEBPA	5
FABP4	5
CEBPB	5
CISH	5
GHR	5
GCG	5
CRLF2	5
HGF	5
CTSS	5
IL2RG	5
MMP3	5
DHCR24	5
DPP4	5
MME	5
EPB42	5

Table S2. Core targets screened from the fospropofol PPI network.

Gene Name	Degree
FN1	28
CD4	28
ITGB1	22
APOA1	20
APOB	20
PRKACB	18
UGT1A1	17
PRKACA	17
PRKACG	16
C3	15
VTN	15
KIT	15
EPO	14
NFKB1	14
SCARB2	14
ERBB2	13
STAT5A	11
STAT5B	11
KDR	11
G6PC	11
SERPINC1	11
ITGB3	10

TFRC	10
AKR1C3	10
GART	10
PLPP2	10
GATA1	9
CD27	9
SREBF1	9
HPX	9
HNF4A	9
C4A	9
ATIC	9
PKLR	9
GATM	9
BCO1	9
MYH7	8
EPOR	8
ITGA9	8
IL6R	8
TNFRSF18	8
TNFRSF14	8
TIGIT	8
LAG3	8
SERPING1	8
F10	8
MTHFD1	8
AR	8
ANPEP	8
CSF1R	8
SLC2A2	8
BHMT	8
AGXT	8
ADCY9	8
ADCY6	8
NPPA	7
CTSS	7
HGF	7
THBS2	7
TNFRSF4	7
TNFRSF8	7
F7	7
KLKB1	7
FABP1	7
HKDC1	7
PDE10A	7
SGPP2	7
CERS3	7
CERS2	7
SLC9A3	6
SULT2A1	6
PC	6
F9	6
SERPIND1	6

GABARAPL1	6
CTSL	6
COL6A3	6
FABP4	6
CDH17	6
SLC12A1	6
ATP1A1	6
GLUL	6
CD68	6
ARG1	6
NR0B2	6
LCAT	6
PTGS2	6
ALDOB	6
HSD3B2	6
HSD3B1	6
AKR1B10	6
SPHK2	6
ACAN	6

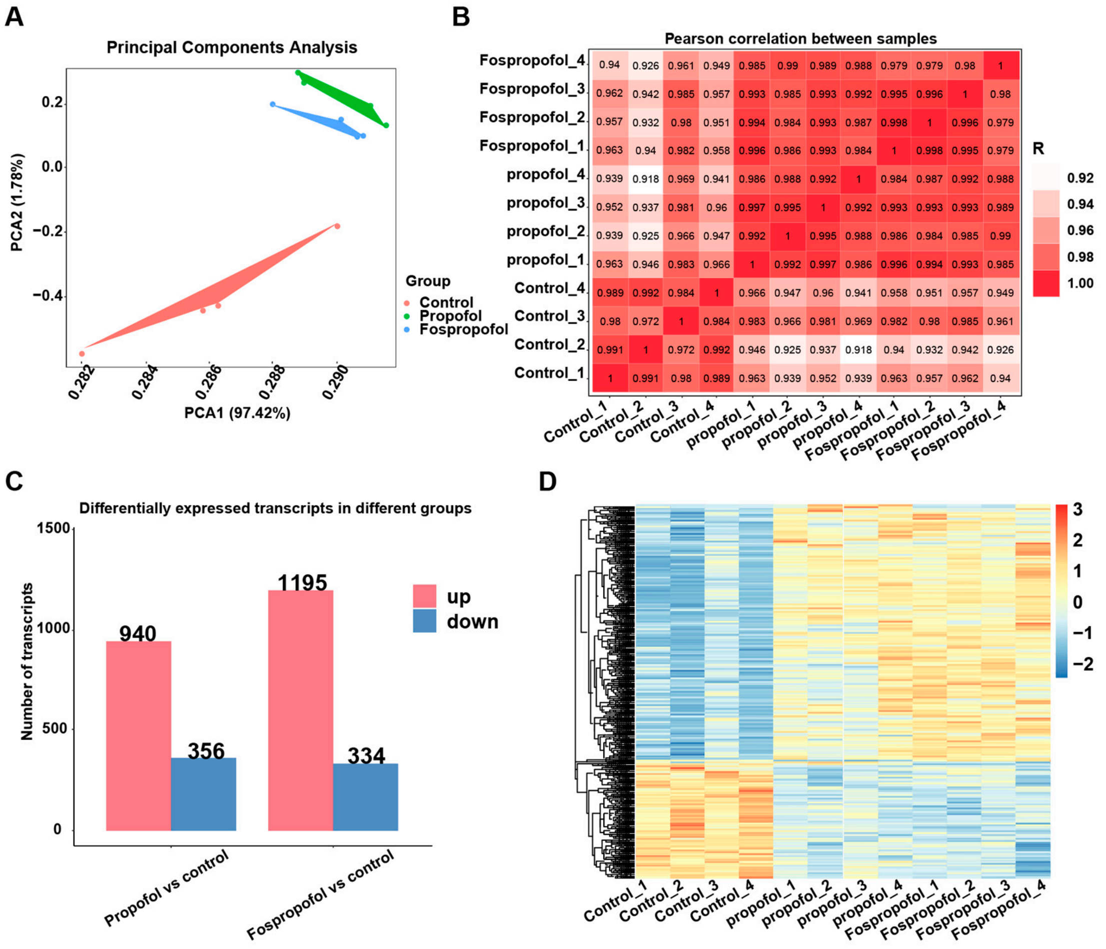

Figure S1. Data Quality Assessment and Differential Gene Expression Analysis. (**A**) Principal component analysis (PCA) plot. (**B**) Pearson correlation coefficients between biological replicates. (**C**) Number of differentially expressed genes (DEGs) per treatment group. (**D**) Hierarchical clustering heatmap of differentially expressed genes (DEGs).

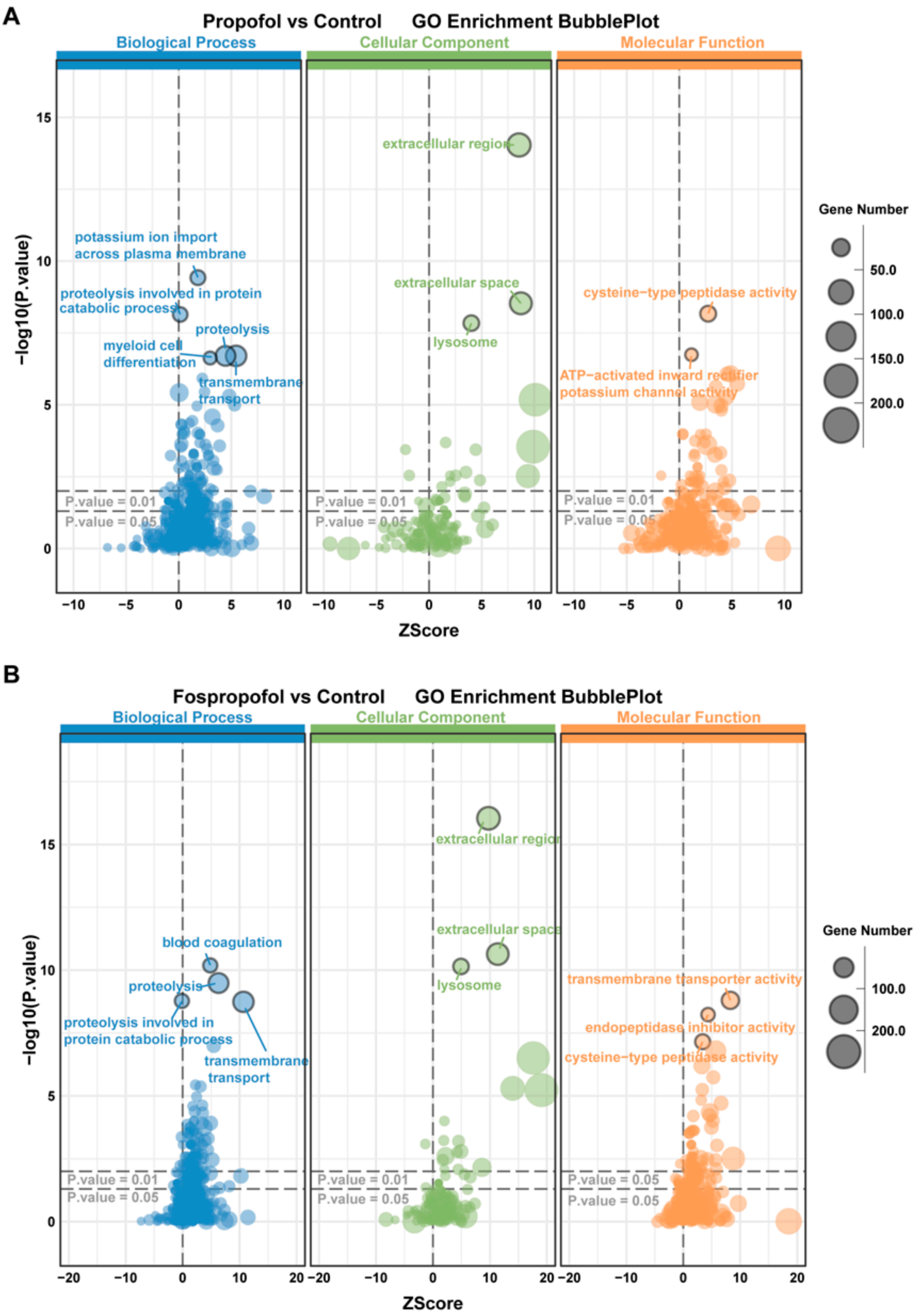

Figure S2. Gene Ontology (GO) pathway enrichment analyses revealed molecular mechanisms underlying propofol and fospropofol-induced effects in zebrafish. (**A**) GO enrichment results for the propofol group. (**B**) GO enrichment results for the fospropofol group.
